# Deficit in Motor Training-Induced Clustering, but Not Stabilization, of New Dendritic Spines in *fmr1* Knock-Out Mice

**DOI:** 10.1371/journal.pone.0126572

**Published:** 2015-05-07

**Authors:** Benjamin C. Reiner, Anna Dunaevsky

**Affiliations:** Department of Developmental Neuroscience, Munroe-Meyer Institute, University of Nebraska Medical Center, Omaha, Nebraska, United States of America; CNRS UMR7275, FRANCE

## Abstract

Fragile X Syndrome is the most common inherited intellectual disability, and Fragile X Syndrome patients often exhibit motor and learning deficits. It was previously shown that the *fmr1* knock-out mice, a common mouse model of Fragile X Syndrome, recapitulates this motor learning deficit and that the deficit is associated with altered plasticity of dendritic spines. Here, we investigated the motor learning-induced turnover, stabilization and clustering of dendritic spines in the *fmr1* knock-out mouse using a single forelimb reaching task and *in vivo* multiphoton imaging. We report that *fmr1* knock-out mice have deficits in motor learning-induced changes in dendritic spine turnover and new dendritic spine clustering, but not the motor learning-induced long-term stabilization of new dendritic spines. These results suggest that a failure to establish the proper synaptic connections in both number and location, but not the stabilization of the connections that are formed, contributes to the motor learning deficit seen in the *fmr1* knock-out mouse.

## Introduction

Fragile X Syndrome (FXS) is the most common inherited intellectual disability [1]. In 95% of cases, FXS is caused by a trinucleotide repeat expansion in the 5’ untranslated region of the *FMR1* gene [2]. This leads to transcriptional level silencing and a complete lack of the *FMR1* gene product, Fragile X Mental Retardation Protein (FMRP) [3]. FMRP is a RNA binding protein thought to be involved in translational down-regulation and synaptic plasticity [4–6]. Children with FXS only display subtle changes in their physical characteristics [7], leading to mild motor and/or language delays being the first diagnosable symptoms [8]. Normal motor skill development is important for exploration, imitation [9] and communication [10], all areas children with FXS struggle.

The *FMR1* gene is highly conserved across species [11], and the murine FMRP protein has 97% amino acid sequence homology to the human protein [12]. The number of trinucleotide repeats in the 5’ region of the mouse *fmr1* gene is stable across generations, therefore mice do not have the naturally occurring silencing of the *fmr1* gene that leads to FXS in humans. However, targeted deletion of exon 5 of the mouse *fmr1* gene produces a KO mouse lacking in FMRP [13]. This *fmr1* KO mouse shows physical and behavioral characteristics observed in humans with FXS [14–17], including a mild motor learning deficit [18].

Motor skill learning is known to involve changes in synaptic plasticity in the primary motor cortex (M1). It has been shown to increase synaptic strength in intracortical connections of the M1 [19, 20], occlude induction of long-term potentiation (LTP, [19, 21, 22]) and induce changes in dendritic spines [23–25]. More specifically, motor skill learning has been shown to induce the stabilization [25] and clustering [23] of newly formed dendritic spines. The spine changes are likely to be mediated by an LTP-like mechanism [20], as LTP has been shown to induce the formation of new dendritic spines near recently active dendritic spines [26]. In the *fmr1* KO mouse, LTP is impaired in several brain regions [27–32], including the motor cortex [18]. Additionally, motor training-induced synaptic changes including synaptic strengthening, occlusion of LTP, synaptic GluA1 insertion and dendritic spine formation are also impaired [18]. What is not known is whether *fmr1* KO mice have deficits in the stabilization and clustered formation of new dendritic spines. To investigate this, we utilized repeated *in vivo* multi-photon microscopy during motor skill training of *fmr1* KO and wild-type littermate mice and quantified the stabilization and clustering of newly formed dendritic spines. We report that in addition to the previously described impairment in formation of new spines, *fmr1* KO mice have a deficit in motor learning-induced new dendritic spine clustering, but no deficit in the specific stabilization of newly formed dendritic spines.

## Materials and Methods

### Animals

All animals were cared for in accordance with NIH guidelines and all experiments were approved by the University of Nebraska Medical Center Institutional Animal Care and Use Committee. C57Bl/6 female heterozygous *fmr1* KO mice [13] were crossed with Tg(Thy1-YFP)H Jrs/J males (The Jackson Laboratory). Male YFP-expressing *fmr1* KO and wild-type littermate offspring were used for all experiments.

### Motor skill training

Methods for motor skill training have been previously described [18]. Briefly, P35–42 mice were food deprived overnight to achieve 85% of their free feeding weight. Mice were placed in a clear training box with a small slit in one wall, and trained to reach and grasp food pellets using their preferred forelimb. A 4 mm gap between the platform holding the food pellet and the slit in the wall of the training box prevented mice from sliding the food pellets towards themselves. An initial pretraining session was used to determine the forelimb preference, and pellets were placed so that they could only be retrieved by the preferred forelimb for all subsequent training sessions. Mice underwent one training session per day for five days. Training sessions last for 100 reach attempts or 30 minutes, whichever came first. Motor skill performance was quantified as the percentage of successful retrievals (number of successfully retrievals divided by the total number of attempts). The trained (tr) hemisphere is contralateral to the trained forelimb, and the untrained (utr) hemisphere is ipsilateral to the trained forelimb.

### Cranial Windows

The technique for cranial window preparation has been previously described [18]. Briefly, thinned-skull cranial windows were prepared over the forelimb region of the M1 of one hemisphere of each animal. Rectangular windows measuring 1.5 mm wide by 1.0 mm long were prepared with the corner closest to Bregma being positioned 750 μm laterally and 0 μm anteriorly. Mice were anesthetized with a ketamine/dexdomitor cocktail (100 mg/ml and 0.5 mg/ml respectively, 2.5 ml/Kg), and boosters were given when necessary, as assessed by pedal reflex. After a midline incision, the skull was thinned using a high speed dental drill, stopping frequently to apply a saline cooling solution. Final thickness was achieved by hand thinning using a dental microblade (Surgistar). A custom made metal head bar was glued to the skull to allow attachment to a custom microscope stage, and was removed after each imaging session. The thinned skull cranial window was covered by suturing the skin between imaging sessions, and the sutures were removed and the window was rethinned before subsequent imaging days. Mice were revived from anesthesia with Antisedan (atipamezole hydrochloride 5.0 mg/ml, 0.2 mg/ml ketamine/dexdomitor cocktail used). Animals were administered carprofen (Pfizer, 0.75 mg) injections daily.

### Multiphoton Imaging

**Fig 1:**
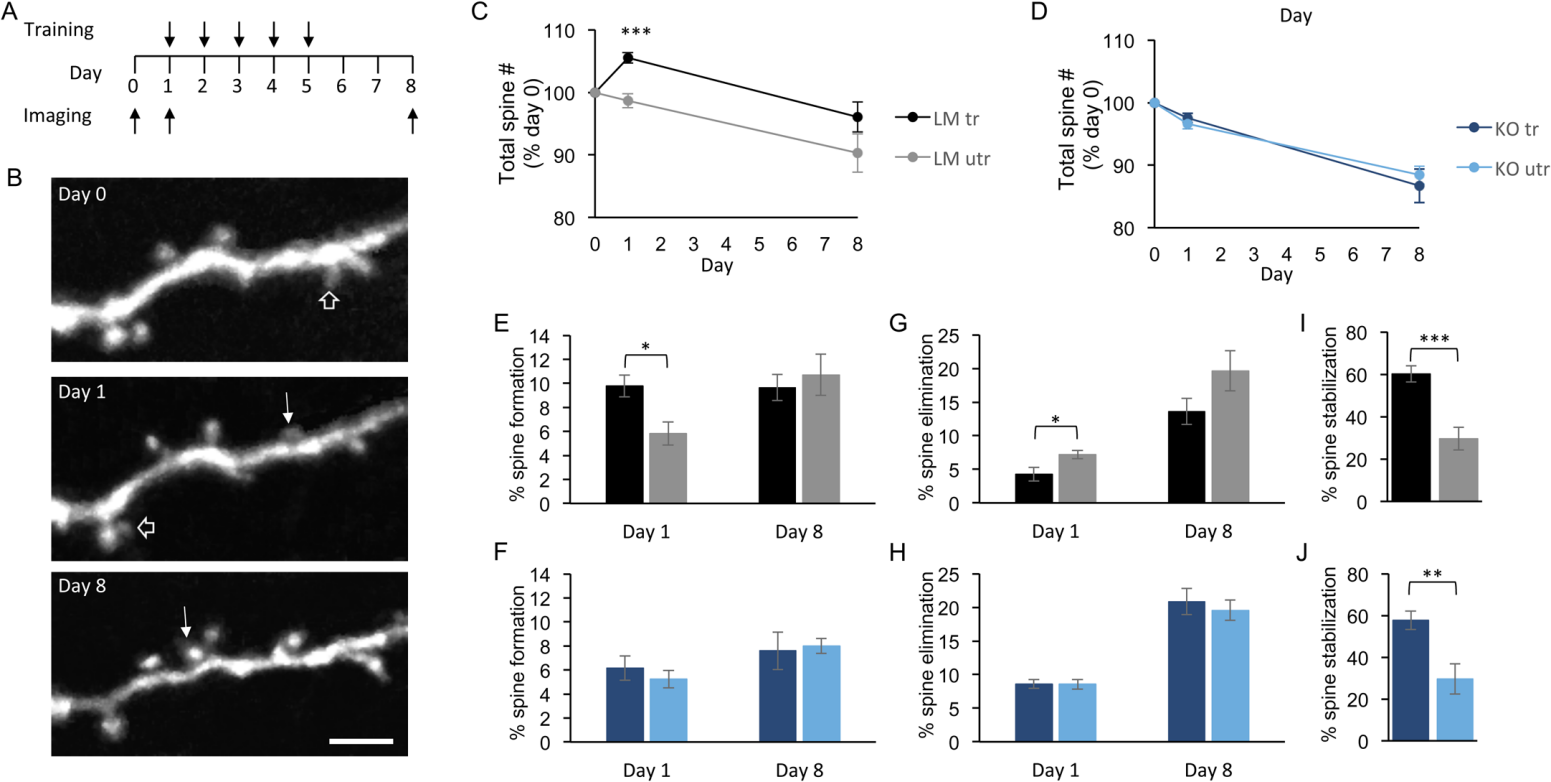
Normal motor learning-induced dendritic spine stabilization in *fmr1* KO mice. (A) Timeline of motor skill training and *in vivo* imaging. (B) Example *in vivo* multiphoton images of layer 1 dendritic spines from a trained hemisphere of a wild-type mouse. Newly formed and eliminated dendritic spines are marked with solid and open arrows respectively. (C) Wild-type littermate mice had a temporary increase in the total number of dendritic spines in the trained hemisphere after a single session of motor skill training, while (D) *fmr1* KO mice have no motor skill training-induced difference in the total number of dendritic spines. Wild-type littermates had significant motor learning-induced changes in dendritic spine formation (E) and elimination (G) on day 1, but not day 8. *Fmr1* KO mice had no motor training-induced changes in the rate of dendritic spine formation (F) or elimination (H) on day 1 or day 8. However, motor skill training did increase the stabilization of newly formed dendritic spines in the trained hemisphere of wild-type littermate (I) and *fmr1* KO (J) mice. Scale Bar = 5 μm, * p < 0.05, ** p < 0.01, *** p < 0.001.

Procedures for *in vivo* multiphoton imaging have been previously described [18]. Imaging was performed using a Movable Objective Microscope (MOM, Sutter), using a Ti:sapphire laser (Chameleon Vision II, Coherent) tuned to 925 nm. A Nikon 60X, 1.0 NA water-immersion lens was used for image collection. Excitation power was adjusted using a Pockels cell to produce near identical levels of fluorescence during each imaging session, and power at the back aperture was typically ≈30 mW. We utilized Scanimage software [33] written in MATLAB (MathWorks) for image acquisition. Four to six regions of interest were collected per animal, and the XYZ coordinators from the motor controller were recorded for reidentification on subsequent imaging days. Images were collected within layer 1 as stacks of 20–80 optical sections separated by 0.48 μm axially at 512 x 512 pixels, 0.13 μm/pixel. Imaging sessions took place two hours after pretraining or motor skill training for imaging days zero and one, and at approximately the same time of day for imaging day eight (see timeline, Fig 1A). For presentation purposes, images were despeckled and axons traversing the field of view were removed from some planes prior to maximum intensity projection.

### Analysis

Dendritic spines were analyzed using ImageJ. Dendritic segments were identified in three-dimensional image stacks at different time points. Dendritic protrusions were identified by scrolling through individual z-planes within a stack, and protrusions were marked and numbered. Dendritic filopodia were identified as long dendritic protrusions with no head, as previously described [18], and were excluded from analysis. Images were compared to that of the initial imaging session. The number of dendritic spines listed for each experiment is the number of spines on Day 0 for which the fate could be determined. This number becomes smaller over time, because not all regions of interest can be reidentified at later imaging time points and are therefore excluded from the calculations. Dendritic spines were considered stable when they appeared in both the initial image and the one being analyzed, eliminated when they appeared in the initial image but not the one being analyzed and newly formed when they appeared in the image being analyzed but not the initial image. Dendritic spine elimination and formation rates were calculated by comparing the number of eliminated or newly formed dendritic spines to the number of dendritic spines present in the initial image, respectively. Specific stabilization of dendritic spines was calculated as the percentage of dendritic spines that were newly formed after the first motor skill training session, that were still present in the final imaging session. Clustering analysis was performed by measuring the distance between dendritic spines that were newly formed after the first motor training session. Newly formed dendritic spines that were within 5 μm of each other were considered to be clustered, regardless if other dendritic spines were found in between [23].

### Statistics

Data is reported as mean ± SEM, and n equals mice. Normality was tested using the Shapiro Wilk test. Analysis was done using two-sided unpaired Student’s t-test.

## Results

### *fmr1* KO mice have normal motor learning-induced stabilization of new dendritic spines

Motor skill learning has been shown to increase the formation and stabilization of new dendritic spines in the trained (tr) hemisphere of wild-type mice [18, 25]. We have recently shown that *fmr1* KO mice have a deficit in the motor learning-induced increase in new dendritic spine formation seen in wild-type mice [18], but the effect of motor skill learning on the stabilization of the dendritic spines that are formed in the *fmr1* KO mouse remains unknown. To investigate this, we trained *fmr1* KO mice expressing YFP in a subset of layer 5 pyramidal neurons and wild-type littermate (LM) mice on a single forelimb-reaching task for five days and imaged dendritic spines in layer 1 of the forelimb region of the M1 *in vivo* (Fig 1B) using multiphoton laser scanning microscopy (see timeline, Fig 1A). We find that, as previously reported [18, 25], wild-type littermate mice have an increase in the total number of dendritic spines in the trained hemisphere after one day of motor skill training, as compared to the untrained (utr) hemisphere (LM tr: 105.52 ± 0.86%, 743 spines, n = 7 mice; LM utr: 98.66 ± 1.14%, 640 spines, n = 6 mice, p = 0.002, Fig 1C). This increase in the total number of dendritic spines was driven by a significant increase in formation of new dendritic spines (LM tr: 9.79 ± 0.91%; LM utr 5.83 ± 0.96%, p = 0.0103, Fig 1E) and a significant decrease in the elimination of preexisting dendritic spines (LM tr: 4.27 ± 1.01%; LM utr: 7.17 ± 0.59%, p = 0.0373, Fig 1G). When the trained and untrained hemispheres were compared on day eight, the increase in the total number of dendritic spines in the trained hemisphere was no longer observed (LM tr: 96.06 ± 2.39%, 665 spines, n = 7 mice; LM utr: 90.31 ± 3.07%, 530 spines, n = 6 mice, p = 0.16, Fig 1C). There was also no difference in the rate of new dendritic spine formation (LM tr: 9.65 ± 1.08%; LM utr: 10.71 ± 1.72%, p = 0.59, Fig 1E) or preexisting dendritic spine elimination (LM tr: 13.59 ± 1.94%; LM utr: 19.66 ± 3.00%, p = 0.10, Fig 1G).

In *fmr1* KO mice, there was no increase in the total number of dendritic spines in the trained hemisphere after one day of motor skill training (KO tr: 97.57 ± 0.75%, 556 spines, n = 6 mice; KO utr: 96.66 ± 0.86%, 705 spines, n = 9 mice, p = 0.47, Fig 1D), consistent with a previous report [18]. As expected, there was no significant difference in the level of dendritic spine formation (KO tr: 6.16 ± 1.01%; KO utr: 5.22 ± 0.73%, p = 0.45, Fig 1F) or elimination (KO tr: 8.59 ± 0.66%; KO utr: 8.56 ± 0.72%, p = 0.97, Fig 1H). When imaged on day eight, no difference was observed in the total number (KO tr: 86.71 ± 2.69%, 492 spines, n = 6 mice; KO utr: 88.42 ± 1.44, 495 spines, n = 7 mice, p = 0.57, Fig 1D), formation rate (KO tr: 7.58 ± 1.55%; KO utr: 7.99 ± 0.61%, p = 0.59, Fig 1F) or elimination rate (KO tr: 20.87 ± 1.97%; KO utr: 19.57 ± 1.50%, p = 0.10, Fig 1H) of dendritic spines. We next asked if the newly formed spines in the trained hemisphere were more likely to be stabilized than newly formed spines in the untrained hemisphere. As expected, littermate mice had a significant increase in the stabilization of new dendritic spines that were formed after a single motor training session, over eight days in the trained hemisphere (LM tr: 60.14 ± 3.75%; LM utr: 29.58 ± 5.30%, p = 0.0005, Fig 1I). To our surprise, *fmr1* KO mice also had a significant increase in the level of new dendritic spine stabilization in the trained hemisphere (KO tr: 57.70 ± 4.35%; KO utr: 29.60 ± 7.32%, p = 0.0091, Fig 1J), which was comparable to the proportion of newly formed spines stabilized in the trained hemisphere in the wild-type littermates. These experiments suggest that the primary deficit is in motor training-induced dendritic spine formation rather than stabilization.

### *fmr1* KO mice have a deficit in the motor learning-induced clustering of new dendritic spines

**Fig 2:**
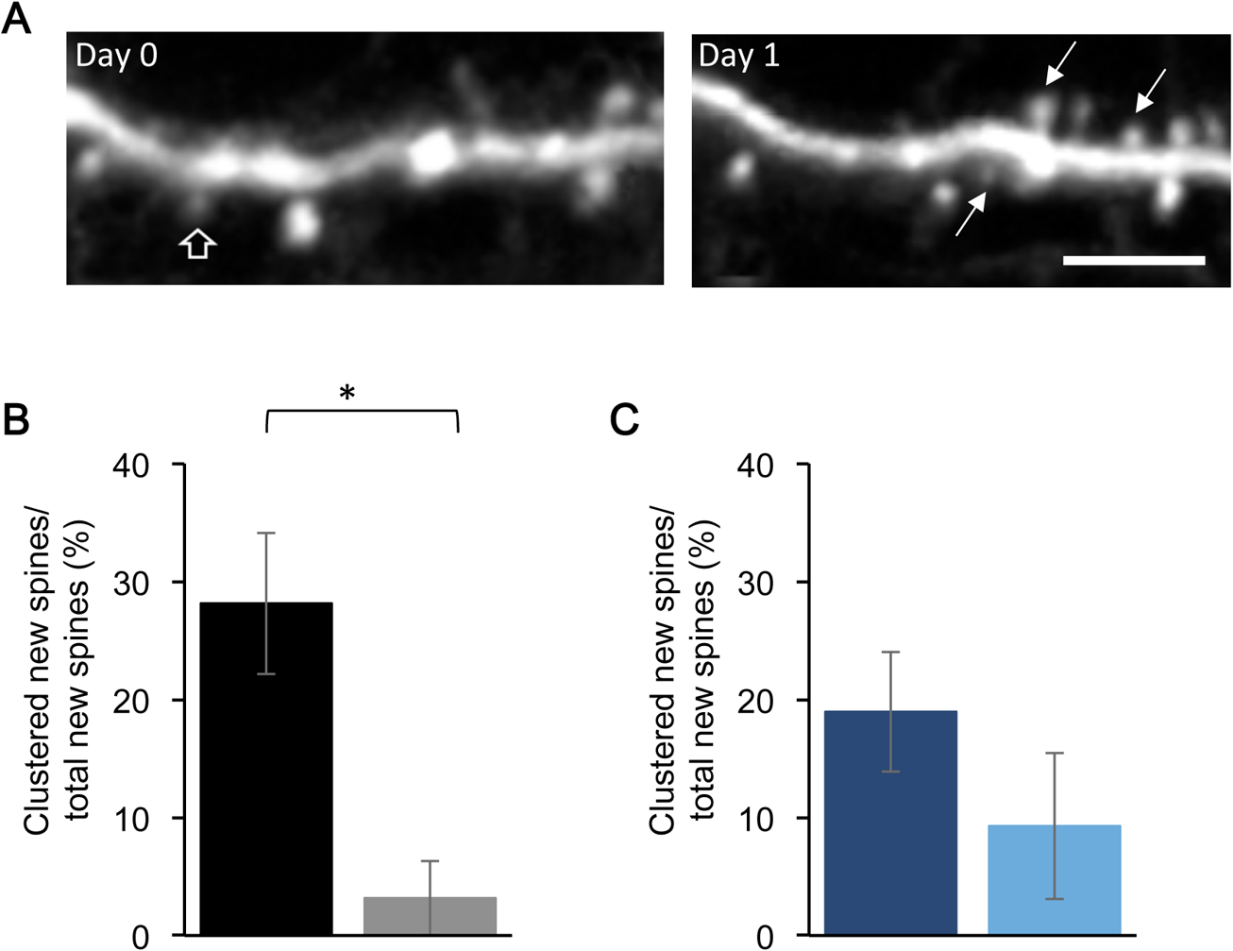
Deficit in motor skill training-induced clustering of new dendritic spines in *fmr1* KO mice. (A) Example of training-induced clustered formation of new dendritic spines. Spines were considered clustered when they formed less than 5 μm apart (arrows). (B) Wild-type littermate mice had a motor training-induced increase in the clustering of new dendritic spines in the trained hemisphere, while *fmr1* KO (C) mice have no difference in the rate of motor skill training-induced dendritic spine clustering in the trained hemisphere. Scale Bar = 5 μm, * p < 0.05.

Previously, it has been shown the motor learning-induced dendritic spines formation is not random, but rather is clustered [23]. New spines are more likely to form next to other new spines in the trained M1. Knowing that *fmr1* KO mice have a mild motor learning deficit and impairments in motor learning-induced dendritic spine structural plasticity [18], we wanted to investigate whether *fmr1* KO mice also had a deficit in the motor training-induced clustering of new dendritic spines. To do this, we imaged dendritic spines after a single motor skill training session, and measured the distance between new dendritic spines. New dendritic spines that were within 5 μm of each other were considered clustered, regardless of other spines being in between. For this analysis we combined the group of mice used for Fig 1 with additional mice that were only imaged for two days. We find that wild-type littermate mice have an increase in the motor training-inducing clustering of new dendritic spines after a single motor training session (LM tr: 28.16 ± 5.99%, 1959 spines, 185 new spines on Day 1, n = 16 mice; LM non-trained 3.17 ± 3.17%, 1977 spines, 111 new spines on Day 1, n = 15 mice, p = 0.0147, Fig 2B). However, *fmr1* KO mice did not show a training-induced increase in dendritic spine clustering (KO tr: 18.97 ± 5.07%, 2052 spines, 140 new spines on Day 1, n = 13 mice; KO non-trained: 9.29 ± 6.21%, 1280 spines, 89 new spines on Day 1, n = 10 mice, p = 0.25, Fig 2C). These experiments indicate that not only are fewer new dendritic spines formed after a single session of motor skill training in the *fmr1* KO mouse, but the clustered spatial organization of the newly formed spines is also impaired.

## Discussion

This study examined the motor training-induced dynamics of dendritic spines in the *fmr1* KO mouse that might be contributing to the motor learning deficit observed in this mouse model of FXS. Here, we confirmed the previously described increase in the formation rate of new dendritic spines after a single day of motor skill training and specific stabilization of these newly formed spines over eight days in wild-type littermate mice [25]. We also saw a significant decrease in the elimination of preexisting dendritic spines in the trained hemisphere of the wild-type littermate mice after one day of motor skill training. The training-induced alterations in spine formation and elimination were transient and no longer observed after 8 days. These results differ from a previous report [25], possibly due to differences in the design of the control condition. Rather than comparing the trained hemisphere of a trained animal with a hemisphere in a non-trained animal [25], here we compared the trained hemisphere of a trained animal with the untrained hemisphere of a trained animal as the control [18]. The decrease in the total number of dendritic spines over eight days in the untrained hemisphere of the wild-type littermates and the trained and untrained hemispheres of the *fmr1* KO mice is similar to a previous report [25] and is likely due to the normal developmental decrease in overall synapse number at this age [34].

In agreement with our previous study [18], we observed no change in the rate of formation or elimination of dendritic spines after one day of motor skill training in the *fmr1* KO mice. The inability of the motor training task to modulate changes in dendritic spines is similar to a previous report that turnover of dendritic spines in *fmr1* KO mice is unaffected by the modulation of sensory experience [35]. Interestingly, *fmr1* KO mice stabilized new dendritic spines that were formed after a single motor skill training session over eight days at a rate almost identical to that of the wild-type littermates. This result was surprising, as we and others had previously determined that basal levels of spine formation and elimination were higher in the *fmr1* KO mice [18, 35, 36]. This suggests that as in the WT mice, the dendritic-spines formed during motor skill training are different in their molecular composition and/or activation and are therefore preferentially stabilized. Taken together, these results imply that while *fmr1* KO mice have a deficit establishing new synaptic connections with motor learning, they are fully capable of stabilizing the connections that are formed. While the *fmr1* KO mice do improve their performance on the motor reaching task over time, they never reach the success rate seen in the wild-type littermate mice [18]. We therefore suggest that the deficit in the motor learning-induced new spine formation, at least in part, underlies the deficit in motor learning seen in the *fmr1* KO mouse. Conversely, the smaller number of motor learning-induced new dendritic spines that are stabilized in the *fmr1* KO mouse likely represent part of the refinement of the enduring motor memory engram that produces the improvements in motor skill performance that are seen in the *fmr1* KO mice. This also implies that the mechanism(s) underlying the rapid increase in new spine formation seen after a single session of motor skill training may be independent of that of long-term synapse stabilization.

The clustered plasticity model suggests synapses that are closer together along the same dendrite are more likely to transmit similar information, compared to synapses that are dispersed over greater distances [37, 38]. This hypothesis is supported by experiments showing that LTP induction promotes the formation of new dendritic spines, and that these newly formed functional connections are likely to form closer to activated preexisting dendritic spines [26]. Moreover, clustering of activated connections leads to strengthening and stabilization of the preexisting synapse [26]. The correlation between the formation of the new dendritic spine and the strengthening of the preexisting synapse suggest that both are likely to be participating in the same neuronal circuit. Additionally, it has been shown that motor learning is able to induce the clustered formation of new dendritic spines in the trained hemisphere of wild-type mice, and that these clustered synaptic connections are preferentially stabilized over time [23]. This implies that the clustered connections are induced by the repetitive activation of the cortical circuits involved in motor learning, and that their preferential stabilization may provide a structural basis for the motor memory of the learning task [23]. The LTP-induced clustering and synaptic strengthening likely applies to the encoding of motor memory, as motor learning has been shown to use an LTP-like mechanism [21, 22]. In comparison to a previous study, where the clustering of new dendritic spines was shown over multiple days of motor training [23], here we detected clustering of new dendritic spines over a single session of motor skill training in wild-type littermates. The fact that we saw similar results over different time frames is likely due to the differences in experimental conditions of the single forelimb-reaching tasks. While in the previous study mice only trained for up to 30 attempts of the task per day, in this study mice performed up to 100 repetitions in the single session of the task (with an average of 48 attempts). In a single training session, mice were more likely able to activate the cortical circuits utilized in motor learning and therefore drive the clustered formation of the new dendritic spines.

Knowing that *fmr1* KO mice have deficits in motor learning and the induction of LTP, both shown to induce the clustered formation of synaptic connections, we postulated that *fmr1* KO mice might also have a deficit in the clustering of motor training-induced new dendritic spines. One possible outcome could have been that the new dendritic spines that are being formed during motor skill training in the *fmr1* KO mouse, although reduced in total number, could still form clusters. Yet what we observed was that *fmr1* KO mice do not show the motor training-induced increase in the clustering of new dendritic spines in the trained hemisphere. This deficit is not due to a difference in the performance of the single forelimb-reaching task, as the *fmr1* KO mice on average perform the same number of attempts and achieve the same success rate as the wild-type littermates over a single training session [18]. One possible explanation for the lack of dendritic spine clustering may simply be that not enough new dendritic spines are being formed for the clusters to appear. Alternatively, the lack of motor learning-induced clustering seen in the *fmr1* KO mice may imply a deficit in the underlying mechanism(s). Since LTP induces the clustering and stabilization of dendritic spines [26], and *fmr1* KO mice have a deficit in LTP induction [18] as well as exaggerated long-term depression [39], it’s likely that the changes in functional plasticity seen in *fmr1* KO mice underlie the impairments in the dendritic spine structural plasticity reported here.

LTP-induced [40] and experience dependent AMPA receptor subunit (GluA1) trafficking into clustered synapses has been previously demonstrated [41]. Activity-dependent GluA1 insertion into synapses has been shown to be impaired in neuronal cultures made from *fmr1* KO mice [42]. Moreover, we have previously reported that GluA1 insertion into synapses with motor skill learning was impaired in the *fmr1* KO mouse [18]. Nevertheless, we think that GluA1 insertion in unlikely to mediate the deficit in clustered formation of spines we observe after a single session of motor skill training, because it occurs at a slower time scale. While the increase in clustered spines was observed two hours post training, motor learning-induced GluA1 insertion does not occur until 18 hours post training [18]. Nevertheless, a cooperative increase in stability and functional plasticity seen in clustered new dendritic spines is believed to require messenger RNA transport and local protein synthesis in dendritic spines [26], processes known to be regulated by FMRP [5, 43]. The lack of motor learning-induced clustering likely represents a deficit in the refinement of the motor engram necessary to optimally perform the reaching task, and may contribute to the reduced motor learning seen in the *fmr1* KO mouse.
